# Renal cortex blood perfusion contrast‐enhanced ultrasound: Assisting diagnosis and treatment of renal artery disease

**DOI:** 10.1002/agm2.12355

**Published:** 2024-08-14

**Authors:** Guo Yaming, Chen Zuoguan, Li Yongjun, Zhang Wayne W

**Affiliations:** ^1^ Department of Vascular Surgery, Beijing Hospital, National Center of Gerontology, Institute of Geriatric Medicine Chinese Academy of Medical Sciences & Peking Union Medical College Beijing People's Republic of China; ^2^ Division of Vascular and Endovascular Surgery, Department of Surgery University of Washington Seattle Washington USA

## Abstract

Renal cortical blood perfusion CEUS can evaluate the structure and microcirculation of renal cortex, which is expected to provide a safer and more convenient evaluation system for clinicians in the diagnosis and treatment of early renal artery disease.

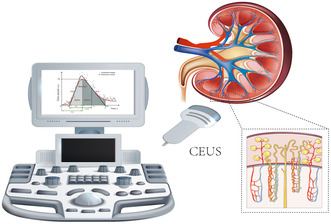

Renal artery disease is a prevalent condition leading to inadequate renal perfusion in clinical practice. The etiology encompasses renal artery atherosclerosis, thrombosis, arteritis, fibromuscular dysplasia (FMD), and dissection. Atherosclerotic stenosis is the primary cause, and the timing of intervention for this type of disease relies heavily on early assessment of renal function. Currently, utilized indicators such as blood creatinine and urine protein may not detect early renal damage. Nuclear renal scintigraphy (NRS) can provide a more sensitive evaluation of renal glomerular filtration function, but its clinical application is limited due to drawbacks such as radiation exposure. Contrast‐enhanced ultrasound (CEUS) involves intravenously injecting microbubble contrast agents that produce enhanced echo signals through vibration or rupture of the microbubbles under the action of ultrasound waves, resulting in clear visualization of tissue structures.^1^ Contrast‐enhanced ultrasound (CEUS) has been widely utilized for the assessment of suspected renal artery stenosis and abnormal luminal structures, as well as to evaluate organ microvascular perfusion.^2^ The recent consensus on methodological standards for cortical perfusion highlights the potential for CEUS to offer standardized procedures and quality control for renal assessment, providing a safer and a more convenient evaluation system for early diagnosis and treatment of renal artery disease. This article provides a brief introduction of CEUS in accessing renal cortex perfusion, along with a brief review of the application and future prospects in renal artery disease.

## 1 RENAL CORTEX BLOOD PERFUSION CEUS AND PARAMETERS

**FIGURE 1:**
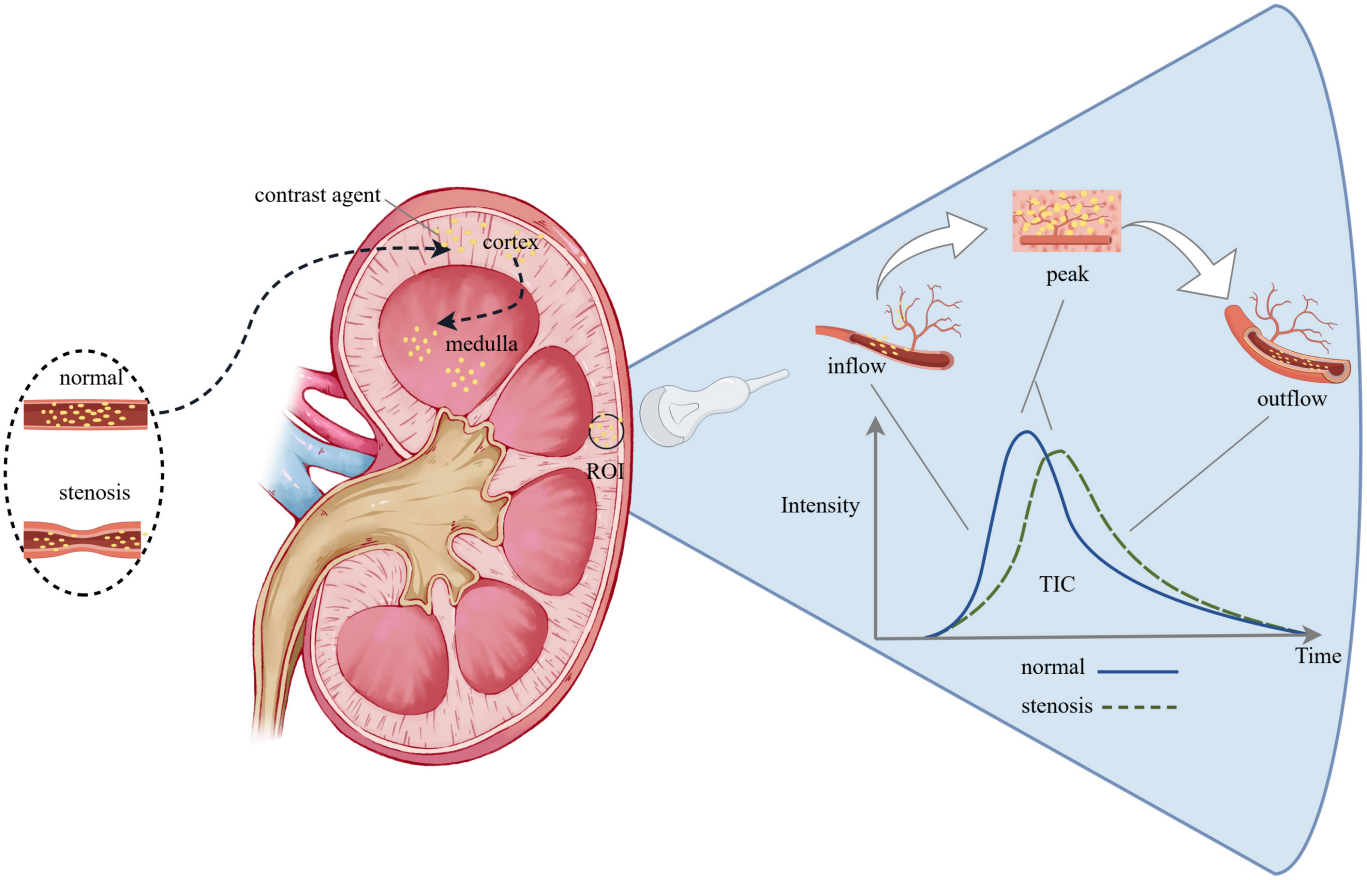
Contrast agent diffusion resulting in the generation of a time‐intensity curve (TIC) within a specific region of interest (ROI).

**FIGURE 2:**
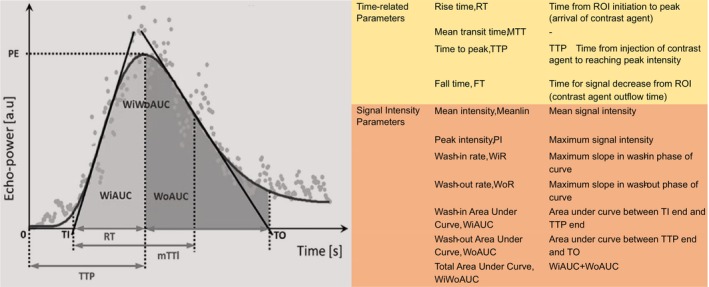
Illustration of the TIC curve and the parameters.

The kidneys are highly vascularized organs, with a rich distribution of cortical blood vessels that provide favorable conditions for perfusion assessment. Renal cortex blood perfusion CEUS enables sequential visualization of perfusion, beginning from the main blood vessels and extending to the small vessels. During the early arterial phase, the renal cortex is initially visualized, followed by the medulla. Corticomedullary differentiation increases, peaks at 20–40 s after injection, then diminishes and disappears within 2 min, resulting in a dynamic change in echo intensity and forming a time‐intensity curve (TIC).^3^ Each point on the TIC curve represents the ultrasound signal intensity at a specific moment. The ascending part of the curve signifies the dispersion of the contrast agent in the bloodstream, while the descending part indicates its clearance (Figure 1).Time‐related parameters and signal intensity‐related parameters reflecting tissue hemodynamic characteristics are derived from the shape and slope of the curve, including those utilized for evaluating renal perfusion (Figure 2). Abnormalities in these parameters can serve as early indicators of renal function and pathological changes, preceding alterations in biochemical markers such as blood creatinine.^4^

## 2 APPLICATION OF RENAL CORTEX BLOOD PERFUSION CEUS IN THE DIAGNOSIS AND TREATMENT OF RENAL ARTERY DISEASES

**FIGURE 3:**
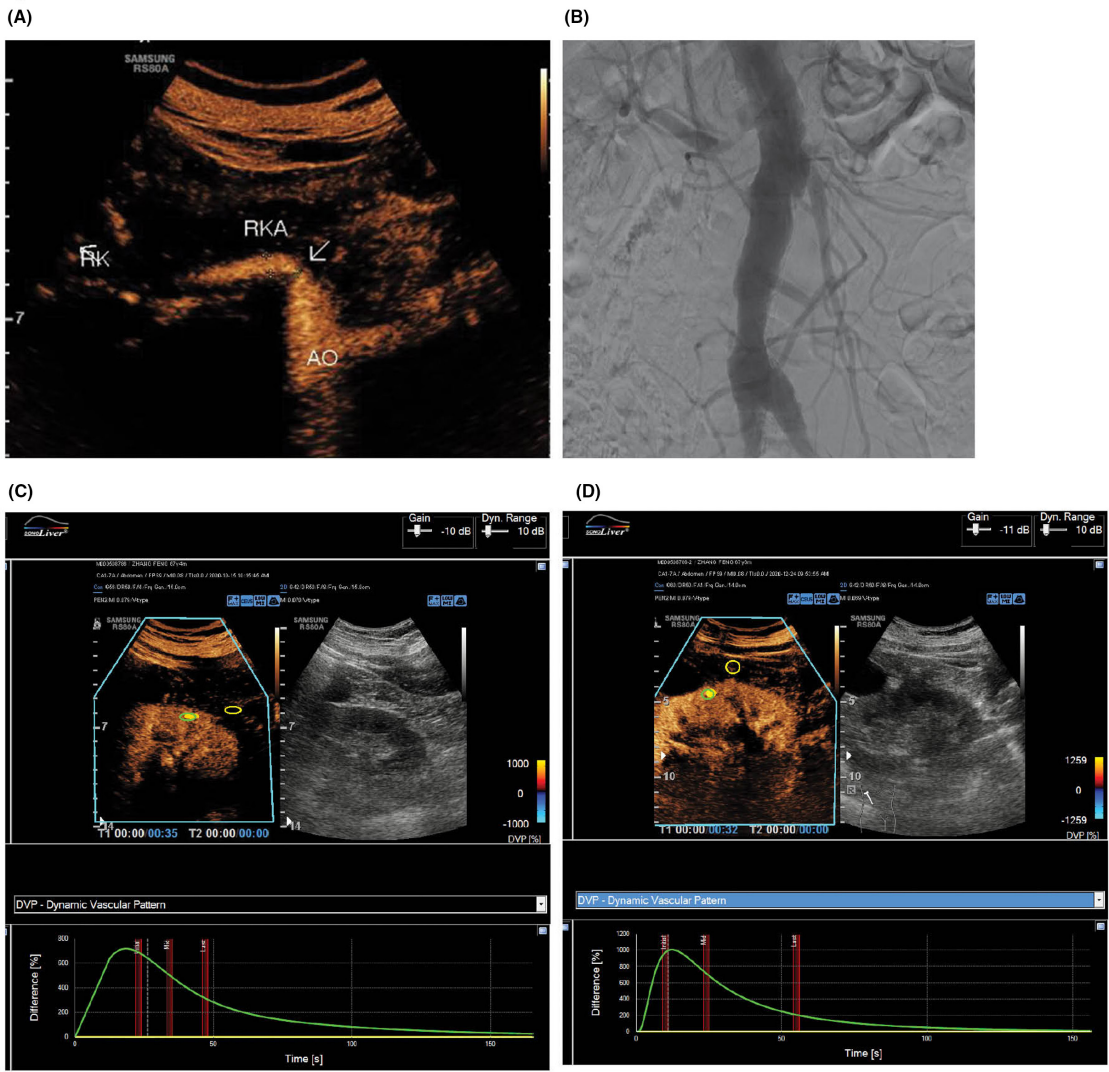
Routine ultrasound and CEUS (A) and DSA (B) showed 85% localized stenosis of the right renal artery with impaired renal cortical blood perfusion (C), which significantly improved after stent implantation (D).^6^

The current guidelines for diagnosing renal artery stenosis recommend renal artery ultrasound as the initial approach, primarily using the peak blood flow velocity to assess the extent of vascular stenosis.^5^ In contrast, CEUS of renal cortex blood perfusion and renal contrast enhanced renal artery ultrasound offer a comprehensive evaluation of the lesion morphology and perfusion, aiding in identifying patients who may benefit from intervention (Figure 3).^6^ In cases of suspected renal infarction, CEUS reveals wedge‐shaped non‐enhancing areas in the infarcted region, clearly distinguishing infarction from cortical ischemic areas, with diagnostic performance comparable to CTA.^7^ Furthermore, parameters of renal cortex blood perfusion can be utilized to assess the prognosis of patients with renal artery stenosis. Ran et al. developed a prognostic model by measuring various parameters of renal artery cortex perfusion combined with machine learning, demonstrating that CEUS scoring of renal cortex blood perfusion is one of the prognostic factors for hypertension following renal artery angioplasty.^8^ In another study, Li and colleagues constructed a prognosis model using data on renal cortex blood perfusion and serum creatinine from 497 patients, revealing that the area under the washout phase AUC is associated with the 1‐year decline in renal function outcome for these patients.^9^

Additionally, the assessment of renal artery stenosis in transplant kidneys presents another suitable application scenario for CEUS. Graft renal artery stenosis typically becomes evident between 3 months and 2 years following renal transplantation, with an incidence as high as 23%.^10^ The AUC value and PI value were found to be effective predictors of the benefits of improving renal function following endovascular treatment for transplant renal artery stenosis.^11^ Compared to the native kidney vasculature, the location of transplant renal arteries is more superficial, which facilitates the use of renal artery CEUS and renal cortex blood perfusion CEUS with less technical challenges. Furthermore, there are no concerns regarding nephrotoxicity associated with digital subtraction angiography (DSA) or CTA contrast agents, making it an ideal method for monitoring perioperative renal function in transplant kidneys.^12^

## 3 ADVANTAGES AND LIMITATIONS OF RENAL CORTEX BLOOD PERFUSION CEUS

The primary benefit of renal cortex blood perfusion CEUS lies in its early diagnostic capability. In addition, it is safe and non‐invasive. Unlike radiotracers, iodine agents, and gadolinium agents, ultrasonic imaging intensifiers avoid the adverse impact of these drugs on the kidneys and reduce the incidence of allergic reactions.^5^ Furthermore, it provides a comprehensive assessment, as it can be combined with renal artery contrast‐enhanced ultrasound for simultaneous evaluation of renal artery structure. Lastly, CEUS is efficient and convenience, enabling bedside examinations with shorter exam times, particularly beneficial for elderly patients who may not be suitable for MRI exams or have mobility issues. Moreover, it helps reduce excessive medical costs for patients and provides convenience for follow‐up and clinical care.

Despite these advantages, CEUS also has inherent limitations compared to traditional ultrasound. First, in obese patients, factors such as susceptibility to respiratory motion, abdominal fat, intestinal gas, and rib shadows may compromise image quality. Second, it may produce false positives or false negatives in the presence of complex, tortuous, or variant lesions. Additionally, unlike CT or MRI which offer comprehensive imaging capabilities, ultrasound measurements are limited to single‐layer sections and may not fully capture the overall renal cortex blood flow situation. Consequently, values obtained from different sections may result in poor reproducibility of parameters. Almushayt's study suggest that time‐related parameters have more diagnostic significance than intensity‐related parameters.^13^ Furthermore, current guidelines do not provide reference ranges for different parameters, which is particularly important for patients with bilateral renal lesions. For patients with unilateral renal lesions, normal reference ranges are often derived from the contralateral normal kidney. This variability is due to differences in parameter definitions based on different instrument settings and analysis platforms, as well as a lack of sufficient research correlating these parameters with renal biopsy results or renal eGFR levels to determine the optimal guiding parameters.

## 4 PROSPECTS

Compared to traditional ultrasound and other imaging modalities such as CTA or MRA, the efficient and convenient advantages of CEUS provide crucial support for its widespread application in the early diagnosis and treatment of renal vascular diseases. With continuous technological advancements, renal cortex CEUS can serve as a more sophisticated, valuable, and safe non‐invasive imaging tool, offering essential information on renal artery structure and microcirculation for clinicians to make informed decisions. It still holds promising prospects for application in the following areas: (1) Supplementary information for intervention indications in renal artery stenosis is essential. Currently, guidelines primarily rely on peak systolic velocity (PSV) values from Doppler ultrasound for intervention indications of renal artery stenosis. As the research content of this technology continues to expand, it may effectively complement existing guidelines for intervention indications in patients with renal artery stenosis. (2) Evaluation and treatment guidance for complex renal vascular diseases is crucial. In complex renal vascular disorders such as abdominal aortic aneurysm or dissection involving renal arteries, kidney perfusion plays a critical role in patient prognosis. If renal cortex blood perfusion CEUS can provide more detailed clinical information for perioperative reconstruction strategies and renal function assessment in these cases, patients will benefit significantly.

Future technological advancements are anticipated to further mitigate current limitations. First, the development of microbubble contrast agents tailored for specific organs holds the potential to extend imaging duration and eliminate the need for repeated injections. For example, a liver‐specific ultrasound contrast agent has already demonstrated prolonged imaging times and significantly enhanced spatial resolution for small lesions.^14^ Similarly, kidney‐specific contrast agents in development are expected to facilitate more comprehensive and sequential assessments of both kidneys simultaneously. Second, the integration of deep learning and artificial intelligence (AI) is projected to leverage CEUS data to refine diagnostic models.^15^ These algorithms may also enable the fusion of images from various scanning modalities, thus offering a more holistic evaluation of renal structures and aiding in the determination of optimal reference parameters. In the future, such advancements are expected to significantly enhance early screening, diagnosis, preoperative evaluation, and intraoperative monitoring of clinical renal artery stenosis.

In conclusion, renal cortex blood perfusion CEUS shows promising application prospects in the diagnosis and treatment of renal artery diseases, providing important clinical guidance for vascular surgeons. Further clinical study in the related field is needed to establish a solid foundation for the optimal utilization of this technology in management of renal artery diseases.

## AUTHOR CONTRIBUTIONS

Yongjun Li conceptualized the editorial. Yaming Guo and Zuoguan Chen wrote the manuscript, Wayne W. Zhang reviewed and edited the manuscript. All authors approved the definitive version of the manuscript.

## FUNDING INFORMATION

This work was financially supported by CAMS Innovation Fund for Medical Sciences (CIFMS‐2021‐I2M‐C&T‐095) AND PUMC Discipline Construction Project (No. 201920102101).

## CONFLICT OF INTEREST STATEMENT

The authors declare that there are no potential conflicts of interest. All authors have reviewed and agreed to the final statement.

## ETHICS STATEMENT

Not Applicable.
